# PorinPredict: *In Silico* Identification of OprD Loss from WGS Data for Improved Genotype-Phenotype Predictions of P. aeruginosa Carbapenem Resistance

**DOI:** 10.1128/spectrum.03588-22

**Published:** 2023-01-30

**Authors:** Michael Biggel, Sophia Johler, Tim Roloff, Sarah Tschudin-Sutter, Stefano Bassetti, Martin Siegemund, Adrian Egli, Roger Stephan, Helena M. B. Seth-Smith

**Affiliations:** a Institute for Food Safety and Hygiene, Vetsuisse Faculty, University of Zurich, Zurich, Switzerland; b Infectious Diseases and Hospital Epidemiology, University Hospital Basel, Basel, Switzerland; c Internal Medicine, University Hospital Basel, Basel, Switzerland; d Intensive Care Unit, University Hospital Basel, Basel, Switzerland; e Division of Clinical Bacteriology and Mycology, University Hospital Basel, Basel, Switzerland; f Applied Microbiology Research, Department of Biomedicine, University of Basel, Basel, Switzerland; g Institute of Medical Microbiology, University of Zurich, Zurich; Instituto Oswaldo Cruz

**Keywords:** *Pseudomonas aeruginosa*, meropenem resistance, outer membrane porin, inactivating mutations, antimicrobial resistance, whole-genome sequencing, surveillance, carbapenem resistance, genotype-phenotype predictions, outer membrane porin OprD

## Abstract

The increasing integration of genomics into routine clinical diagnostics requires reliable computational tools to identify determinants of antimicrobial resistance (AMR) from whole-genome sequencing data. Here, we developed PorinPredict, a bioinformatic tool that predicts defects of the Pseudomonas aeruginosa outer membrane porin OprD, which are strongly associated with reduced carbapenem susceptibility. PorinPredict relies on a database of intact OprD variants and reports inactivating mutations in the coding or promoter region. PorinPredict was validated against 987 carbapenemase-negative P. aeruginosa genomes, of which OprD loss was predicted for 454 out of 522 (87.0%) meropenem-nonsusceptible and 46 out of 465 (9.9%) meropenem-susceptible isolates. OprD loss was also found to be common among carbapenemase-producing isolates, resulting in even further increased MICs. Chromosomal mutations in quinolone resistance-determining regions and OprD loss commonly co-occurred, likely reflecting the restricted use of carbapenems for multidrug-resistant infections as recommended in antimicrobial stewardship programs. In combination with available AMR gene detection tools, PorinPredict provides a robust and standardized approach to link P. aeruginosa phenotypes to genotypes.

**IMPORTANCE** Pseudomonas aeruginosa is a major cause of multidrug-resistant nosocomial infections. The emergence and spread of clones exhibiting resistance to carbapenems, a class of critical last-line antibiotics, is therefore closely monitored. Carbapenem resistance is frequently mediated by chromosomal mutations that lead to a defective outer membrane porin OprD. Here, we determined the genetic diversity of OprD variants across the P. aeruginosa population and developed PorinPredict, a bioinformatic tool that enables the prediction of OprD loss from whole-genome sequencing data. We show a high correlation between predicted OprD loss and meropenem nonsusceptibility irrespective of the presence of carbapenemases, which are a second widespread determinant of carbapenem resistance. Isolates with resistance determinants to other antibiotics were disproportionally affected by OprD loss, possibly due to an increased exposure to carbapenems. Integration of PorinPredict into genomic surveillance platforms will facilitate a better understanding of the clinical impact of OprD modifications and transmission dynamics of resistant clones.

## INTRODUCTION

Carbapenems are last-line antibiotics reserved for the treatment of severe bacterial infections. The emergence of carbapenem-resistant (CR) pathogens has thus created a major challenge for public health. Of particular concern is the spread of carbapenem-resistant Acinetobacter baumannii (CRAB), *Enterobacteriaceae* (CRE; including Klebsiella pneumoniae, Escherichia coli, and Enterobacter spp.), and Pseudomonas aeruginosa (CRPsA), which are major causes of nosocomial and multidrug-resistant infections and associated with high mortality rates (1).

In clinical CRE and CRAB isolates, carbapenem resistance is typically conferred by plasmid-acquired carbapenem-hydrolyzing enzymes (carbapenemases) (2, 3). In contrast, among clinical CRPsA isolates, carbapenemase positivity rates vary considerably between survey sites: rates are below 5% in most European countries and in North America, while rates of 30% to 70% have been reported from studies in Asia (4–11). Among these carbapenemases, VIM and IMP show the highest prevalence; others such as FIM, GES, GIM, KPC, OXA-48, and SPM are often associated with specific geographic regions or high-risk clones, i.e., clones associated with multidrug resistance and global spread (12).

In carbapenemase-negative CRPsA, carbapenem resistance is primarily associated with the inactivation of the OprD outer membrane porin (11, 13). OprD, a monomeric 18-stranded β-barrel, is responsible for the passive uptake of basic amino acids and small peptides and serves as a diffusion channel for carbapenems such as meropenem and imipenem, which structurally resemble dipeptides (14, 15). OprD defects can be mediated by indels, recombination events such as the insertion of insertion sequence (IS) elements, nonsense mutations, start- or stop-loss mutations, promoter disruptions, or amino acid substitutions affecting the porin’s pore width, charge, or structural integrity (7, 11, 16). Other genetic determinants contributing to decreased carbapenem susceptibility include the overexpression of multidrug efflux pumps and the overexpression of the intrinsic beta-lactamase AmpC (17, 18).

Pathogen surveillance has greatly benefited from the recent integration of genomics: the timely and accurate identification of pandemic clones and transmission events is critical to guide public health responses (19). Whole-genome sequencing has also become an integral part of antimicrobial resistance (AMR) surveillance, complementing phenotypic antimicrobial resistance testing by providing valuable information on resistance mechanisms and evolutionary events that led to their acquisition (20). Whereas reliable databases and computational tools are available for the detection of carbapenemase genes from whole-genome sequencing data (summarized by Hendriksen et al. [21]), there is a lack of robust and standardized approaches for the detection of porin loss-mediated carbapenem resistance. The identification of the various possible mutations leading to OprD deficiency currently relies on technical expertise and laborious curation and is thus often omitted from routine genotype-phenotype analyses. To address this, we investigated the genetic diversity of OprD in 2,088 P. aeruginosa genomes and compiled a database of intact variants. This was integrated into PorinPredict, a tool that predicts OprD porin loss from whole-genome sequencing data. PorinPredict was validated using 1,124 additional genomes (including 987 carbapenemase-negative genomes) and facilitated considerably improved genotype-phenotype predictions for P. aeruginosa meropenem resistance.

## RESULTS AND DISCUSSION

### Diversity of OprD in P. aeruginosa.

**FIG 1:**
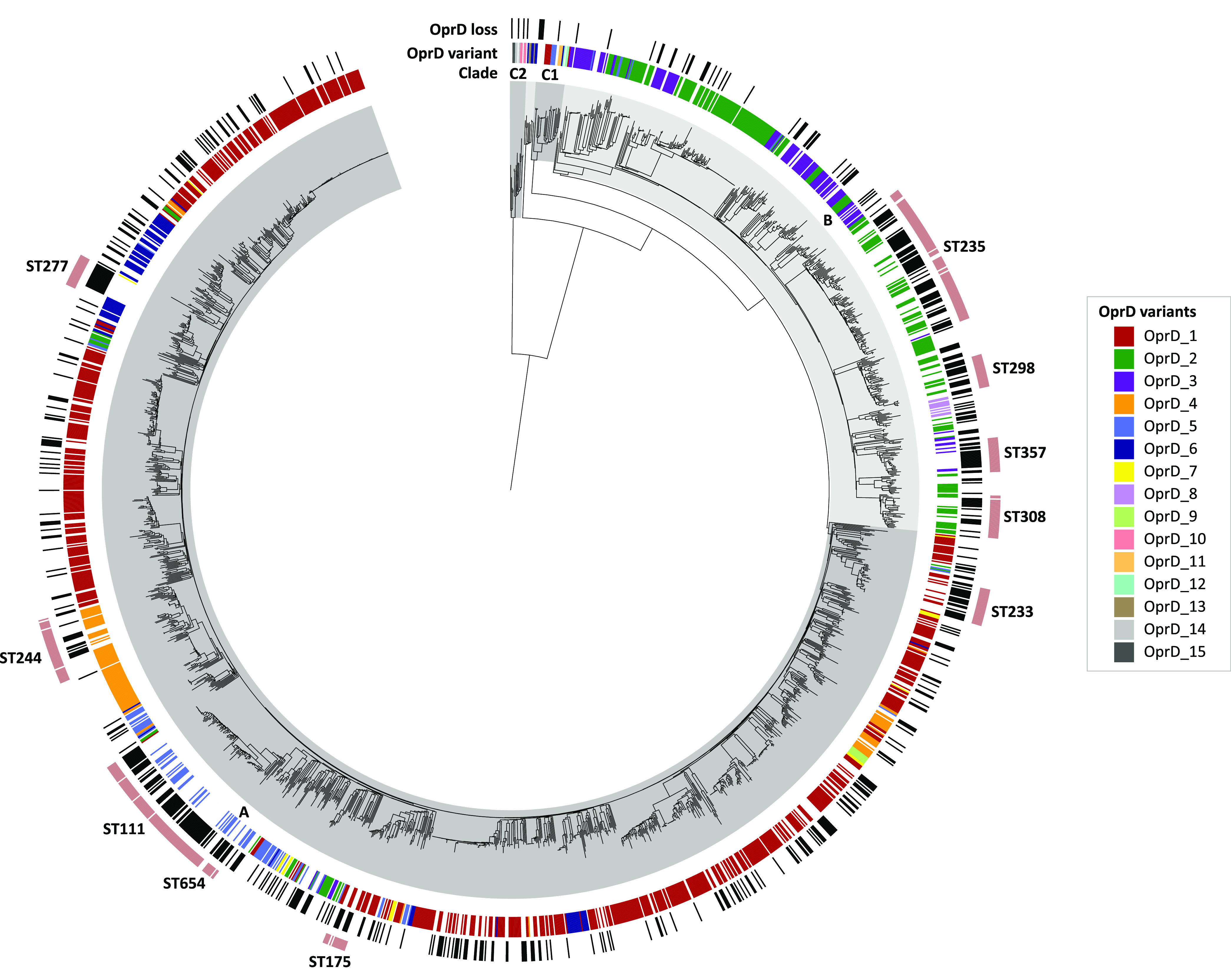
Distribution of OprD variants and predicted OprD loss across the P. aeruginosa phylogeny. Assemblies of 2,088 isolates (NCBI data set) were included in the analysis. Presence of OprD variants (exact amino acid sequence match) is labeled according to the legend. Putative OprD loss is shown in black bars. Intact nonexact matches (*n* = 51) and putative OprD losses due to promoter disruptions (*n* = 12) are not indicated. Isolate affiliation to clades (shaded in gray) and high-risk clones (outermost ring) is annotated. Phylogenetic distances were estimated using MASH and visualized in iTOL (46).

A total of 2,088 high-quality genome assemblies of P. aeruginosa isolates (NCBI data set) covering the phylogenetic clades A (*n* = 1,500), B (*n* = 541), C1 (*n* = 25), and C2 (*n* = 14) and a C1-C2 intermediate clade (*n* = 8) (Fig. 1) were screened for *oprD*. The *oprD* gene or fragments thereof were detected in 2,087 (99.95%) assemblies. Despite a phylogenetically diverse data set, few OprD protein variants were identified: of 1,483 isolates with a presumptively intact *oprD* open reading frame, 1432 (96.6%) encoded 1 of 15 distinct OprD protein variants (exact match), and 51 (3.4%) encoded one of these 15 dominant variants with 1 to 4 variable amino acid substitutions (no length variation). Eight variants were found in isolates belonging to clade A (OprD_1, OprD_2, OprD_3, OprD_4, OprD_5, OprD_6, OprD_7, and OprD_9) and five variants in clade B (OprD_1, OprD_2, OprD_3, OprD_5, and OprD_8). Six (OprD_10 to OprD_15) of the 15 intact variants were only detected in clade C (Fig. 1; see Table S1 in the supplemental material).

**FIG 2:**
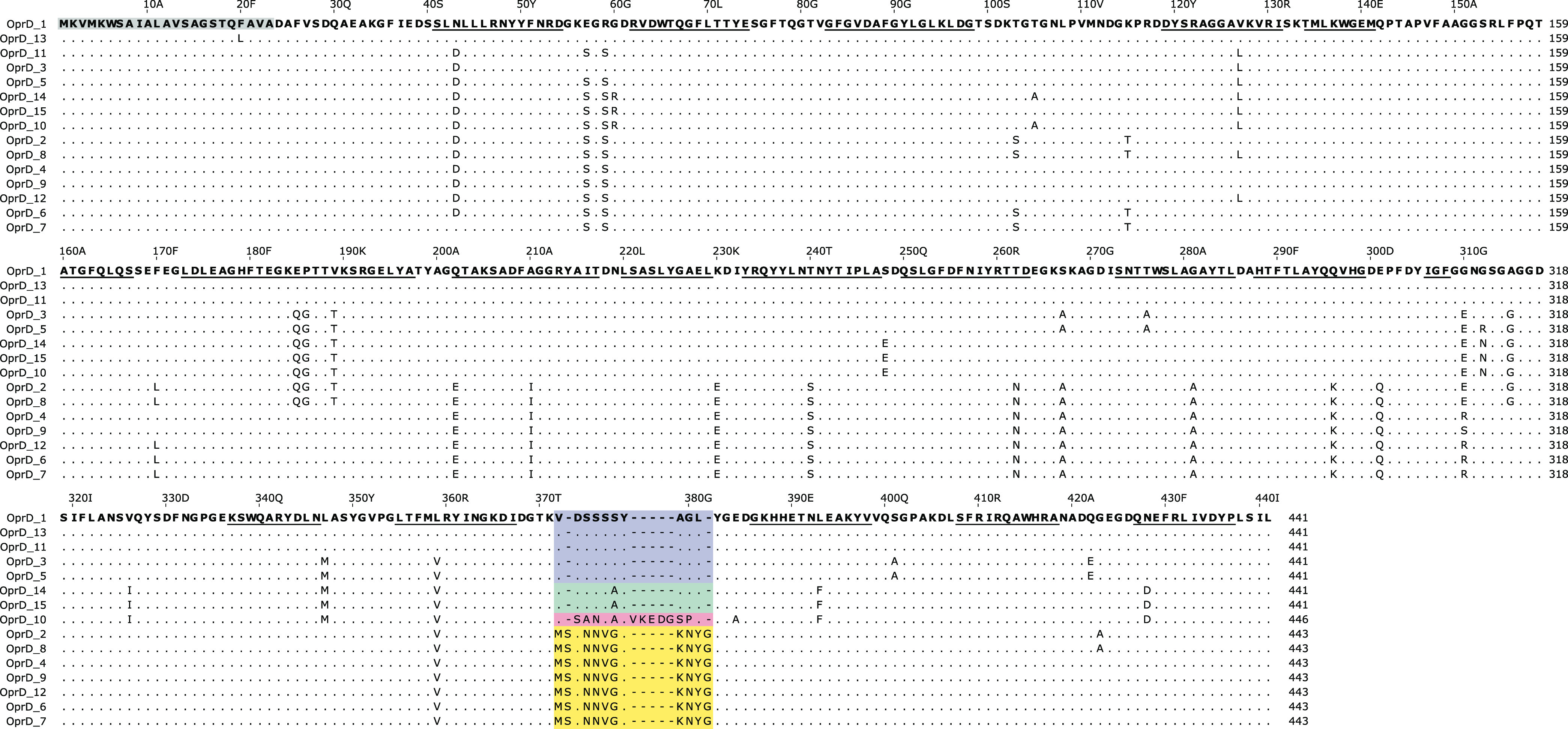
Alignment of P. aeruginosa OprD variants. The variable region within loop 8 is colored (L8_10_, blue; L8_10b_, green; L8_12_, yellow; L8_15_, red). Scale numbers above the alignment refer to amino acid positions in variant OprD_1 and include the signal peptide (23 amino acids, gray box). β-Strands (according to PDB structure 3SY7) are underlined.

The 15 OprD variants consisted of 441, 443, or 446 amino acids and contained one of four C-terminal loop L8 (according to references 15 and 22; formerly L7) sequences, which determined the variants’ overall length, including the previously described L8_10_ (formerly L7_10_; 372-VDSSSSYAGL-381) and L8_12_ (formerly L7_12_; 372-MSDNNVGYKNYG-383) (23), as well as two novel L8 regions detected in clade C2 isolates, L8_15_ (372-VSANSAYVKEDGSPL-386) and L8_10b_ (372-VDSSSAYAGL-381) (Fig. 2). The latter differed from L8_10_ by only one amino acid substitution (S377A). Isolates with the L8_10_ type OprD were previously associated with hypersusceptibility to meropenem, but not to imipenem (23, 24). OprD variants of the same L8 group were polyphyletically distributed across the P. aeruginosa population: both L8_10_- and L8_12_-containing variants were detected in isolates of clades A, B, C1, and the C1-C2 intermediate (Fig. 1).

In the 605 genomes of the NCBI data set with a presumptively inactivated OprD coding sequence, the open reading frame contained premature stop codons (*n* = 150), frameshift mutations (*n* = 297), other indels or truncations (*n* = 155), start-loss mutations (*n* = 2), or was not detected (*n* = 1). Disrupted promoter regions (but intact OprD coding sequences) were identified in nine assemblies, which all contained contig breaks within 200 bp upstream of *oprD*. In all nine cases, sequences matching with various IS elements (IS*1394*, IS*Pa82*, IS*Ppu1*, IS*Ppu21*, IS*Psme1*, IS*Psp2*, IS*Pst7*, or IS*5* family) were identified between the contig break and *oprD* (Table S3). IS elements typically occur in multiple copies in a genome, preventing a resolved assembly of their surrounding region from short-read sequencing data.

The high proportion (28.9%) of genomes with an inactivated OprD coding sequence in this data set likely reflects an overrepresentation of sequenced clinical isolates which had been exposed to antibiotics. Of the isolates, 391 (18.7%) belonged to the high-risk clones sequence type 111 (ST111), ST175, ST233, ST235, ST244, ST277, ST298, ST308, ST357, or ST654, which are known for their increased virulence and multidrug resistance, often associated with various carbapenemases and extended-spectrum beta-lactamases (12). Overall, 239 (61.1%) of all high-risk clone isolates were predicted to be OprD deficient, compared to 375 of 1,697 (22.1%) isolates not belonging to high-risk clones (Fig. 1).

### Evaluation of PorinPredict: concordance of predicted OprD loss and carbapenem resistance.

PorinPredict detects putative OprD defects by querying genomes against a database consisting of the 15 identified intact OprD variants and against an *oprD* promoter database. Truncated or absent *oprD*, indels, premature stop codons, stop-loss and start-loss mutations, and disrupted promoter sequences are reported as putative OprD porin loss. Missense mutations are reported but not *per se* classified as porin loss.

**FIG 3:**
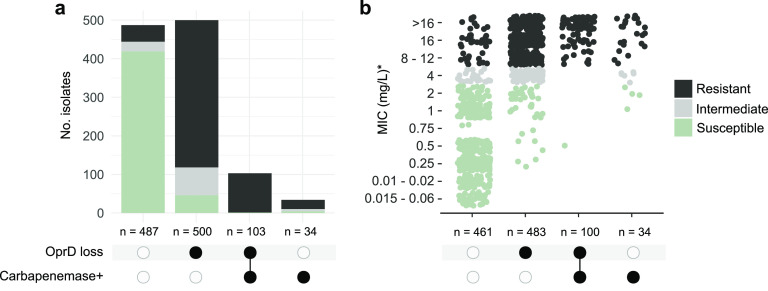
Correlation between predicted OprD loss, presence of carbapenemases, and meropenem resistance phenotype. (a) Intersection of P. aeruginosa isolates with (black circles) or without (white circles) carbapenemase and/or a disrupted OprD. Assemblies of 1,124 isolates (validation data set) were included in the analysis. (b) Approximations of meropenem MICs among a subset of 1,078 isolates with reported values. Due to differing test ranges, MIC values reported as 512, 128, 64, >32, ≥32, 24, or >16 mg/L are here summarized as >16 mg/L; >8 or ≥16 mg/L are shown as 16 mg/L, and ≤0.25 mg/L and <0.25 mg/L are shown as 0.25 mg/L and 0.1 and 0.02 mg/L, respectively, thus representing an approximation.

To evaluate PorinPredict regarding genotype-phenotype associations, 1,124 genomes of P. aeruginosa isolates with known phenotypic meropenem susceptibility (validation data set) were screened for a putative OprD loss and the presence of carbapenemase-encoding genes. OprD loss was common and strongly associated with meropenem resistance: OprD loss was predicted for 556 of 653 (85.1%) nonsusceptible isolates (including 484 of 551 [87.8%] resistant and 72 of 102 [70.5%] intermediately resistant isolates), as well as 47 of 471 (10.0%) susceptible isolates (Fig. 3; Table S2). Carbapenemase-encoding genes were identified in 132 (20.2%) of the nonsusceptible isolates, often (*n* = 102, 83.1%) in combination with an inactivated OprD. Midlevel resistance conferred by carbapenemases may predispose for OprD loss, synergistically decreasing carbapenem susceptibility: isolates with concomitant OprD loss and carbapenemase presence showed a median meropenem MIC of >32 mg/L (interquartile range [IQR], >8 to 128) compared to 12 mg/L (IQR, 4 to >32) among isolates with a carbapenemase alone (Fig. 3b). A similar synergy of carbapenemases and outer membrane porin modifications has been described in carbapenem-resistant K. pneumoniae (25).

In the subset of carbapenemase-negative isolates, OprD loss was predicted for 454 of 522 (87.0%) nonsusceptible and 46 of 465 (9.9%) susceptible isolates (Table 1). Among the 79 isolates obtained at the University Hospital Basel, only 1 (1.3%) isolate contained a carbapenemase; in the remaining (carbapenemase-negative) isolates, OprD loss was predicted for 22 of 23 (95.7%) meropenem-nonsusceptible and 2 of 53 (3.8%) susceptible (MIC = 0.75 mg/L each) isolates.

**TABLE 1:** Genotype-phenotype associations for meropenem resistance and causes of putative OprD loss among 987 carbapenemase-negative P. aeruginosa isolates

Genotype-phenotype association	No. of susceptible isolates (*n* = 465)	No. of intermediately resistant isolates (*n* = 97)	No. of resistant isolates (*n* = 425)
Intact OprD (exact match)^*a*^	408	22	28
Intact OprD (nonexact match)^*a*^	11	3	15
SNAP2 prediction: effect	0	3	12
SNAP2 prediction: neutral	11	0	3
OprD loss	46	72	382
Frameshift mutation	13	33	177
Premature stop	17	22	111
Gene truncation	14	11	87
Other indels	0	2	3
Incomplete promoter^*b*^	2	3	2
Start-loss	0	0	2
Stop-loss	0	1	0

a Intact variants with disrupted promoter regions are counted as OprD loss.

b Counted only if *oprD* coding region was intact.

For a total of 43 (7.8%) meropenem-resistant and 25 (24.5%)-intermediately resistant isolates, the phenotype could not be explained by carbapenemase-encoding genes or OprD loss. However, OprD variants with missense mutations were significantly more common among these isolates (15/43, 34.9% and 3/25, 12.0%) than among meropenem-susceptible isolates with intact OprD (11/419, 2.6%, *P < *0.001, respectively), possibly affecting the structural or functional integrity of OprD (Table 1). Missense mutations in nonsusceptible isolates affected residue R154 (corresponding to R131 when excluding the 23-amino-acid signal sequence), a critical site known to determine pore width (15); H288 and S403, which are both conserved across different members of the OprD family (15); multiple sites in β-strands (S278, Q340, L359) replaced by proline, which disrupts secondary structures; and G316, which forms a hydrogen bond to R391 (R393 in L8_12_), part of the characteristic basic ladder in the OprD pore constriction (15) (Table 2). The R154, G316, S278P, and S403P substitutions were previously associated with carbapenem resistance (16). Using the neural network-based classifier SNAP2, most (15/18) missense mutations in nonsusceptible isolates were predicted to have functional effects (Table 2). Missense mutations in susceptible isolates were typically located in the N-terminal region, more conservative, and predicted to be neutral. The position of the amino acid substitutions in the OprD protein structure is shown in Fig. S1.

**TABLE 2:** Missense mutations in OprD in 29 isolates of the validation data set and associated meropenem susceptibility

Isolate	Meropenem susceptibility	OprD reference variant	Missense mutation(s)	Predicted functional effect (SNAP2)	Expected prediction accuracy (SNAP2 [%])
287.12499_PA	Intermediate	OprD_3	R154H	Effect	95
287.12621_PA	Intermediate	OprD_1	L359P	Effect	75
287.1101_PA	Intermediate	OprD_2	L434R (in L8_12_)	Effect	80
287.5955_PA	Resistant	OprD_11	L127V	Neutral	93
287.12587_PA	Resistant	OprD_4	R154C	Effect	95
287.8031_PA	Resistant	OprD_5	S278P	Effect	91
287.7799_PA	Resistant	OprD_1	S278P	Effect	85
287.1095_PA	Resistant	OprD_5	S278P	Effect	91
287.12616_PA	Resistant	OprD_1	H288R	Effect	85
287.12490_PA	Resistant	OprD_1	L292Q	Effect	80
287.5956_PA	Resistant	OprD_1	G307D	Neutral	57
287.8490_PA	Resistant	OprD_6	G316D	Neutral	61
287.12754_PA	Resistant	OprD_1	G316D	Effect	71
287.12755_PA	Resistant	OprD_1	G316D	Effect	71
287.12669_PA	Resistant	OprD_1	Q340P	Effect	80
287.5971_PA	Resistant	OprD_1	G383D (in L8_10_)	Effect	80
287.12734_PA	Resistant	OprD_2	S403P (in L8_12_)	Effect	63
287.12639_PA	Resistant	OprD_6	L409P (in L8_12_)	Effect	76
287.860_PA	Susceptible	OprD_2	A8S	Neutral	97
502940-8-20	Susceptible	OprD_1	K34N	Neutral	82
502940-9-20	Susceptible	OprD_1	K34N	Neutral	82
502940-3-20	Susceptible	OprD_1	K34N, A247S	Neutral (each)	82, 97
287.7801_PA	Susceptible	OprD_4	D43N	Neutral	95
501539-19	Susceptible	OprD_5	V63A	Neutral	93
500658-19	Susceptible	OprD_6	T66S	Neutral	97
721748-19	Susceptible	OprD_6	T66S	Neutral	97
287.1066_PA	Susceptible	OprD_1	V86I	Neutral	93
287.1043_PA	Susceptible	OprD_2	G98A, V129I	Neutral (each)	82, 97
287.1190_PA	Susceptible	OprD_2	V129I	Neutral	97

OprD loss and carbapenemase-encoding genes were identified in 47 (10.0%) and 5 (1.1%) out of 470 meropenem-susceptible isolates, respectively, and resulted in increased MIC values (median, 2 mg/L) compared to those isolates without any detected carbapenem resistance determinants (median, 0.5 mg/L) (Fig. 3b).

Possible reasons for inconsistent genotype-phenotype associations include mixed bacterial cultures, fragmented or absent (carbapenemase) gene assemblies from short-read data, alternative carbapenem-resistance mechanisms such as mutations affecting the functionality and overexpression of intrinsic beta-lactamases or efflux-pumps, technical errors during the susceptibility testing or sequencing, interlaboratory differences in MIC readings, or transcription errors.

Overall, PorinPredict improved the accuracy of genotype-phenotype predictions for meropenem nonsusceptibility from 53.1% (when predicted based on the presence of carbapenemase-encoding genes alone; sensitivity and specificity of 20.2% and 98.9%, respectively) to 89.6% (when predicted based on presence of carbapenemase-encoding genes and/or porin loss; sensitivity and specificity of 89.1% and 89.4%, respectively). Among the 79 University Hospital Basel (USB) isolates, for which MIC measurements were performed as part of this study, the accuracy was 96.2% (sensitivity and specificity of 95.8% and 96.4%, respectively). Very major discrepancies (resistant isolates predicted to be susceptible) were found for 1 of 21 (4.7%) and 42 of 430 (9.8%) isolates of the USB and PATRIC data sets, respectively. Considering amino acid substitutions at critical sites as inactivating mutations would further improve accuracies and discrepancy rates for genotype-phenotype predictions.

### Role of regulators affecting MexAB and AmpC expression.

Carbapenem susceptibility can be affected by additional genetic factors, including the expression levels of the intrinsic genes *bla*PDC (AmpC beta-lactamase) and *mexA* and *mexB* (MexAB multidrug efflux pump) (17, 18). Here, loss (truncations, premature stop codons, frameshifts, or stop loss) of the MexAB repressors MexR, NalC, and NalD was predominantly found among isolates that simultaneously harbored primary carbapenem resistance determinants (OprD loss, OprD missense mutations, or presence of carbapenemase genes) (Table 3). MexR-, NalC-, and NalD-deficient isolates exhibited 1.5- to 4-fold-increased meropenem MICs compared to those with intact repressor genes. In the absence of carbapenemase genes or OprD loss, these isolates were predominantly classified as susceptible. MexR loss was, however, detected in 11 out of the 43 isolates with very major genotype-phenotype discrepancies, sometimes (*n* = 4) coinciding with OprD missense mutations (Table S2). Loss of the AmpC repressor AmpR (in combination with an intact *bla*PDC) was detected in 20 isolates, which often (*n* = 11; 55.0%) coharbored primary carbapenem resistance determinants. The nine isolates with AmpR loss alone showed no increased MICs (median, 0.5 mg/L; IQR, 0.5 to 1 mg/L). Missense mutations in repressor genes or changes in their binding sites may also affect expression levels but were not further investigated here. Overall, our data suggest that the derepression of efflux pumps and intrinsic beta-lactamase genes plays a minor role in meropenem resistance of clinical P. aeruginosa isolates. Similar results were recently reported for other carbapenems (11).

**TABLE 3:** Effect of defective MexAB repressors on meropenem susceptibility among 1,055 isolates with presumptively intact MexA and MexB

Genotype	Predicted OprD loss, OprD missense mutations, or carbapenemase gene presence	Median MIC (IQR^*a*^ [mg/L])	No. (%) of nonsusceptible isolates
MexR, NalC, and NalD intact (*n* = 878)	Yes (*n* = 501)	8 (8–16)	461 (92.0)
	No (*n* = 377)	0.5 (0.25–1)	38 (10.1)
MexR loss (*n* = 65)	Yes (*n* = 47)	16 (8–32)	40 (85.1)
	No (*n* = 18)	2 (1–8)	7 (38.9)
NalC loss (*n* = 38)	Yes (*n* = 24)	12 (8–28)	22 (91.2)
	No (*n* = 14)	1 (0.25–2.5)	3 (21.4)
NalD loss (*n* = 78)	Yes (*n* = 64)	16 (8–32)	62 (96.7)
	No (*n* = 14)	2 (1.75–2)	2 (14.3)

a IQR, interquartile range.

### Convergent OprD-inactivating mutations and spread of *oprD*-deficient clones.

We observed identical OprD-inactivating mutations in distantly related isolates, suggesting independent acquisitions at vulnerable sites. In the NCBI data set, these included a premature stop at position W277* identified in different OprD variants (OprD_1, OprD_2, OprD_8, and OprD_15) of 28 isolates belonging to 21 distinct (and 3 unknown) sequence types (Table S1). Likewise, the frameshift mutation c.1199_1200insC (in L8_10_ variants [p.Q400fs]) or the corresponding c.1205_1206insC (in L8_12_ variants [p.Q402fs]) was found in multiple OprD variants (OprD_1, OprD_2, OprD_4, and OprD_8) of 38 isolates belonging to at least 22 distinct (and 2 unknown) sequence types.

Identical disruptive mutations were occasionally identified among closely related isolates, pointing to the spread of OprD-deficient clones. A phylogenetic cluster of 18 isolates within ST277 carried the frameshift mutation c.380_381delTG (p.E127fs) within OprD_6 (Table S1). These isolates were obtained between 1997 and 2019 at various geographic locations in Brazil (*n* = 16) and the United States (*n* = 2), corresponding with reports of a Brazilian endemic ST277 clone carrying this stable OprD-inactivating mutation (26). A subclone of the high-risk clone ST175 carrying a characteristic premature stop codon (Q142*) in OprD_1 and detected in Spanish and French hospitals over at least a decade (27, 28) was represented in the validation data set (17 isolates) (Table S2).

### Compensatory mutations and coacquisition of AMR determinants.

The presence of relatively conserved OprD throughout the P. aeruginosa population suggests an important metabolic role of this porin, with OprD loss possibly leading to fitness defects. To investigate potential compensatory mutations acquired by OprD-deficient isolates, we performed a kmer-based genome-wide association study (using DBGWAS) for a subset of 700 isolates with (*n* = 206) versus without (*n* = 494) predicted porin loss (NCBI data set; Table S1). While mutations in *oprD* itself were not captured by DBGWAS, three genetic determinants reached genome-wide significance (*q* [Benjamini-Hochberg adjusted *P*] < 0.05) for associations with OprD-loss, (i) the chromosomal mutation *gyrA* T83I (*q*_min_ = 6.6E-18) and (ii) *parC* S87L (*q*_min_ = 1.6E-7) (both contributing to ciprofloxacin resistance), and (iii) a component (*q*_min_ = 1.4E-5) representing *intI1*, *sul1* (sulfonamide resistance), *qacE*Δ1 (antiseptic resistance), and an acetyltransferase-encoding gene, which are typically colocated on a class I integron (Table S4). Rather than representing compensatory mutations or gene acquisitions, these results likely reflect an accumulation of antimicrobial resistance mechanisms during patient treatment due to the use of carbapenems as a last-resort antibiotic for multidrug-resistant clones, as commonly recommended in antibiotic stewardship programs. In the entire NCBI data set of 2,088 isolates, 65.3% of the OprD-deficient isolates (*n* = 614) carried a mutation linked to quinolone resistance (*gyrA* T83I, *gyrA* D87G, *gyrA* D87H, *gyrA* D87N, *parC* S87L, or *parE* A473V) compared to 18.3% (*P* < 0.001; odds ratio [OR], 8.4; 95% confidence interval [CI, 6.8 to 10.4]) among the isolates with an intact OprD (*n* = 1,474). The *sul1* gene was found in 47.7% of the OprD-deficient isolates, compared to 10.3% (*P* < 0.001; OR, 7.9; 95% [CI, 6.3 to 10.0]) of the isolates with intact OprD.

### Conclusion.

In conclusion, we developed, evaluated, and validated PorinPredict, a much-needed tool that predicts OprD-inactivating mutations in P. aeruginosa. Among carbapenemase-negative isolates, predicted OprD loss was overall highly consistent with phenotypic meropenem nonsusceptibility. Independent prospective validation is, however, necessary to provide evidence suitable for *in vitro* diagnostic regulations (IVDR). Although relatively rare, the effects of OprD missense mutations often remain difficult to interpret. The increasing availability of whole-genome sequencing and associated susceptibility data will enable an extension of the database covering additional variants. Given the increased application of whole-genome sequencing in clinical laboratories, PorinPredict will be valuable for detecting and tracking resistance within patients and communities.

## MATERIALS AND METHODS

### OprD database generation.

For the generation of a database of intact OprD sequences, 2,186 randomly selected P. aeruginosa genome assemblies were retrieved from the NCBI Reference Sequence Database (see “NCBI data set” in Table S1 in the supplemental material), corresponding to approximately 30% of the available RefSeq assemblies (searched in April 2021) and including both clinical and environmental isolates. Species affiliation was confirmed using ribosomal multilocus sequence typing (rMLST) (version 2022-06-23) (29) and sequence types assigned using mlst 2.19.0 (https://github.com/tseemann/mlst). Assembly quality was assessed using QUAST 5.0.2 (30) and CheckM 1.1.3 (31). After removal of low-quality assemblies (*N*_50_ < 15 kb, assembly length < 5.9 Mb, CheckM completeness < 97%, CheckM contamination < 3%), 2,088 assemblies were available to investigate the diversity of OprD. OprD amino acid sequences were extracted using DIAMOND 2.0.7 (32) (reference, GenPept accession no. AKQ14499, BLASTx mode, 70% sequence identity and coverage) and bedtools 2.30.0 (33), and variants were deduplicated using CD-HIT 4.8.1 (34). Deduplicated variants (*n* = 364) with inactivating mutations (*n* = 309; C- or N-terminal deletions; frameshift, nonsense, start-loss, or stop-loss mutations) and rare variants (*n* = 40; identified in <5 isolates of the dominant clades A and B or in <2 isolates of clade C) were excluded. Protein alignments of the remaining 15 intact variants were generated using MAFFT version 7 (35) and visualized using Jalview version 2.11.2.0 (36).

### Development of PorinPredict.

PorinPredict was designed to detect mutations leading to a putative OprD loss or OprD amino acid substitutions from preassembled genomes. PorinPredict uses OprD nucleotide and protein sequence databases in combination with BLASTn 2.5.0+ (37) and DIAMOND 2.0.7 (32), respectively, to assess OprD integrity. BLASTn 2.5.0+ (37) is used with a 95% sequence identity threshold and the options “-max_target_seqs 10 -evalue 1E-20 -culling_limit 1.” The BLASTn hit with the lowest E value is evaluated. DIAMOND is run in BLASTx mode with sequence identity and coverage thresholds of 95% and 60%, respectively, and the options “--query-gencode 11 -b 6 --max-target-seqs 1 --sensitive --masking 0 -c1.” Amino acid substitutions are identified using the R package Biostrings (v3.14) (38). PorinPredict additionally assesses the integrity of the *oprD* promoter region by screening input assemblies against a database consisting of *oprD* promoter regions from five isolates representing the distinct P. aeruginosa clades (A, B, C1, C2, and C1-2 intermediate; defined according to Ozer et al. [39]). These sequences consist of the 200-bp region upstream of *oprD* comprising the ArgR binding site, −35 and −10 region, and Shine-Dalgarno sequence (40) and 10 bp of the N-terminal *oprD* sequence. For the promoter screening, BLASTn 2.5.0+ (37) is used as described above. BLASTn and DIAMOND results are filtered and summarized in an interpretable output table. PorinPredict reports (i) exact matches (same length, no amino acid substitutions) to intact OprD variants in the database; (ii) nonexact matches, i.e., intact same-length variants with amino acid substitutions (missense mutations); (iii) mutations leading to a presumptive OprD loss, including premature stop codons (nonsense mutations), truncations, frameshift mutations and other indels, loss of stop codons (stop-loss), loss of start codons (start-loss), and absence of *oprD* in the assembly (no hit); and (iv) putatively disrupted *oprD* promoter regions (BLASTn matches with <98% coverage).

### Evaluation of PorinPredict.

PorinPredict 1.0.0 was evaluated using a second, independent data set consisting of 1,124 P. aeruginosa genome assemblies that had associated meropenem susceptibility data available and passed the above-defined CheckM and QUAST quality criteria. This collection (“validation data set” in Table S2) included 79 assemblies from isolates of the University Hospital Basel (USB) sequenced as part of this study and 1,045 assemblies accessed from PATRIC (41). For short-read sequencing of in-house isolates, genomic DNA was extracted using the DNeasy blood and tissue kit (Qiagen) and sequenced on the Illumina NextSeq 500 or Illumina MiSeq platforms. Draft genomes were assembled using Unicycler version 0.3.0b (42) after read trimming with Trimmomatic version 0.38 (43).

Meropenem susceptibly of the USB isolates was determined using the microdilution assay Vitek 2 (*n* = 64; bioMérieux) or Etest (*n* = 15; Apteq) (Table S2). Meropenem susceptibility values of the PATRIC data originated from various studies and were determined based on MICs (*n* = 1,001) or disk diffusion (inhibitory zone diameter [IZD]; *n* = 44). When available, MICs and IZDs were interpreted according to CLSI guideline M100 (susceptible, ≤2 mg/L or ≥19 mm; intermediate, 4 mg/L or 16 to 18 mm; resistant, ≥8 mg/L or ≤15 mm) (44). For 29 PATRIC assemblies, only phenotypic classifications (interpreted according to CLSI criteria) but no MIC/IZD values were available (Table S2).

### Genomic analyses.

Phylogenetic distances were estimated using Mashtree 1.2.0 (45) in accurate mode (--mindepth 0), and clades (A, B, C1, C2) were assigned based on phylogenetic clustering with previously typed (39) genomes. Phylogenetic trees were visualized with iTOL version 6 (46). Antimicrobial resistance genes and mutations in quinolone resistance-determining regions were detected using AMRfinder 3.10.24 with default parameters (47). Beta-lactamases were classified as carbapenemases based on antibiotic subclass associations listed in the AMRfinder database. Genome-wide association studies for isolates with versus without predicted OprD loss were performed using DBGWAS version 0.5.4 (options, –maf 0.05 -q100) (48). Due to high computational requirements, the analysis was performed on a subset of 700 randomly selected assemblies of the NCBI data set irrespective of their phylogenetic distribution or susceptibility (Table S4). For selected assemblies, the genetic context of *oprD* was manually investigated in CLC Main Workbench 22.0.1. Insertion sequence (IS) elements were identified using ISFinder (49). Assemblies containing the *oprD* frameshift mutation c.1199_1200insC were identified using BLASTn 2.10.1+ (37) in short-sequence mode with a 38-bp partial *oprD* sequence of NCBI accession nos. USI81034 (intact) and CP050331 (frameshifted) as references. Functional effects of OprD amino acid substitutions were predicted using SNAP2 (50). Assemblies were queried for MexR (references, GenPept accession nos. NP_249115 and WP_003153084), MexA (reference, GenPept accession no. NP_249116), MexB (reference, GenPept accession no. NP_249117), AmpR (references, GenPept accession nos. NP_252798, WP_024915537, and WP_058145575), NalC (references, GenPept accession nos. NP_252410 and WP_033984356), and NalD (reference, GenPept accession no. NP_252264) using DIAMOND 2.0.7 (32) in BLASTx mode (minimum sequence coverage and identity, 50% and 90%, respectively). Assemblies with no hit, incomplete hits (<98% alignment coverage on the amino acid level), or hits with internal stop codons were considered defective for the respective protein. Plots were generated in R 4.0.3 using the ComplexUpset 1.3.3 package (https://zenodo.org/record/5762625#.YrWOVXZByUk).

### Statistical tests.

Frequency counts were compared using a two-tailed Fisher’s exact test. *P* values of <0.05 were considered to reflect statistical significance.

### Data and software availability.

Sequencing data generated as part of this study are available under BioProject accession no. PRJEB54973. Accession numbers of included assemblies are listed in Table S1 (NCBI data set) and Table S2 (validation data set) in the supplemental material. PorinPredict is available under the terms of the GNU General Public License 3.0 at https://github.com/MBiggel/PorinPredict/.
