# Supplementary material for: PorinPredict: In Silico Identification of OprD Loss from WGS Data for Improved Genotype-Phenotype Predictions of P. aeruginosa Carbapenem Resistance
Source: Microbiol Spectr. 2023 Jan 30;11(2):e03588-22. doi: 10.1128/spectrum.03588-22 (PMC10100854; doi:10.1128/spectrum.03588-22)
Supplement: Supplemental file 2 — Supplemental material. Download spectrum.03588-22-s0002.pdf, PDF file, 0.3 MB [file spectrum.03588-22-s0002.pdf]

Supplementary Materials

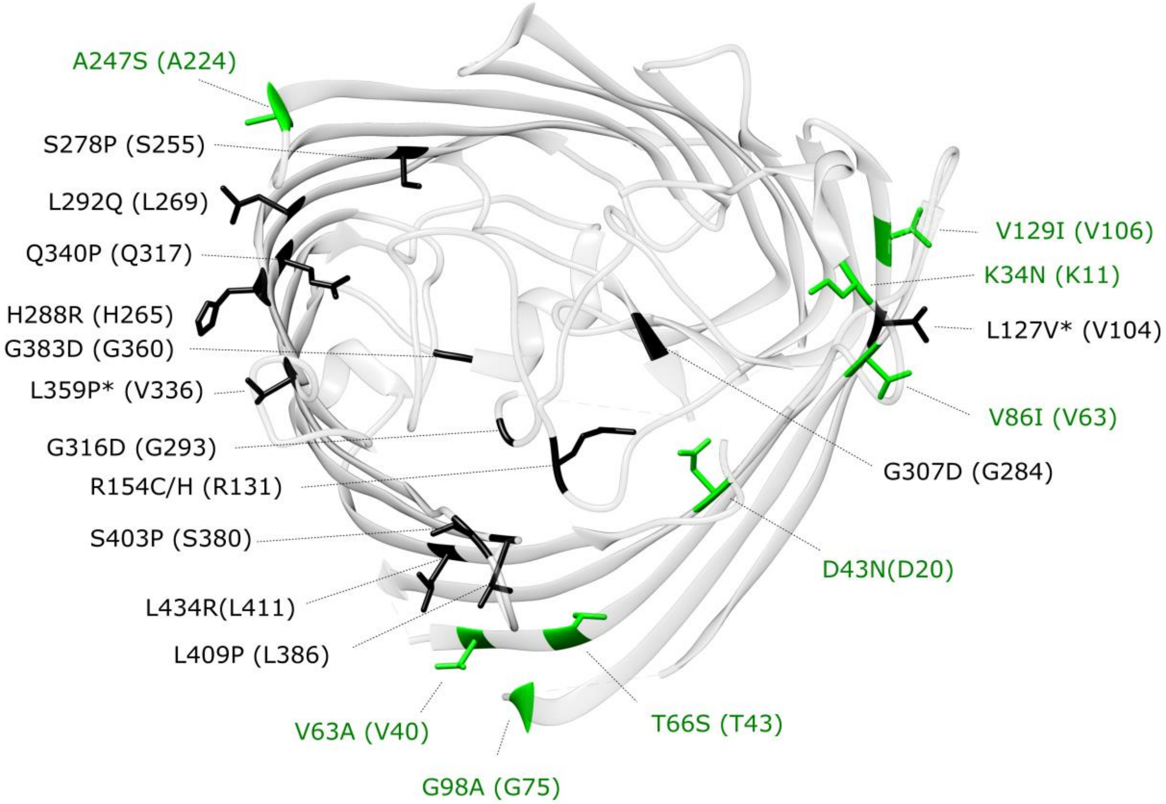

Supplementary Figure 1. Schematic structure of OprD (PDB accession 3SY7). Sites of amino acid substitutions identified in susceptible (green) or non-susceptible (black) carbapenemase-negative isolates of the validation dataset are labelled. Amino acid numbering refers to an $L8_{10}$ type OprD. Numbering without the 23 amino acid signal sequence is given in brackets. In the labelled mutations, wild-type amino acids refer to those of the closest OprD variant in the reference database and may differ from the 3SY7 sequence (asterisk). The image was created using UCSF Chimera software version 1.16 (1).

References

1. Pettersen EF, Goddard TD, Huang CC, Couch GS, Greenblatt DM, Meng EC, Ferrin TE. 2004. UCSF Chimera - a visualization system for exploratory research and analysis. J Comput Chem 25:1605–12.
